# A Home Between Environmental Changes and Climatic Anxiety

**DOI:** 10.3390/ijerph23081038

**Published:** 2026-08-10

**Authors:** Ferrarello Susi

**Affiliations:** Department of Philosophy & Religious Studies, California State University, East Bay, Hayward, CA 94542, USA; susi.ferrarello@csueastbay.edu

**Keywords:** solastalgia, eco-anxiety, emotional geography, phenomenology, culture of emergency, dwelling, Husserl (real/reell), Spielraum, climate-related distress, environmental instability, emotion regulation

## Abstract

**Highlights:**

**Public health relevance—How does this work relate to a public health issue?**

- Solastalgia—the distress of remaining in a place destabilized by environmental change—is a measurable and widespread mental health burden linked to wildfires, floods, drought, and chronic exposure to emergency alerts, affecting populations across the Global North and South.
- The paper shows how a pervasive “culture of emergency”—institutionalized through preparedness drills, meteorological alerts, and crisis protocols—sediments into chronic vigilance and anticipatory anxiety, constituting a public health stressor that operates below the threshold of clinical diagnosis.

**Public health significance—Why is this work of significance to public health?**

- By theorizing solastalgia as a world-relation problem rather than an individual pathology, the paper offers a conceptual framework that reorients climate-related distress away from individualized treatment models and toward shared structural and environmental conditions—with direct implications for how public health responses are designed.
- The interdisciplinary integration of phenomenology, emotional geography, and emotion-regulation theory provides public health researchers and clinicians with a vocabulary for what empirical instruments measure but do not explain: the internal, lived structure of climate-related suffering.

**Public health implications—What are the key implications or messages for practitioners, policy makers and/or researchers in public health?**

- Practitioners addressing climate-related distress should resist framing solastalgia primarily as cognitive distortion or maladaptive appraisal; doing so risks privatizing a structurally induced condition and compounding the isolation it produces. Community-level and intersubjective approaches—mutual aid, shared rituals of grief, and collective efficacy—are integral to restoration.
- Policy makers and researchers must address the material bases of dwelling—including housing stability, equitable access to insurance, and environmental justice—as conditions of mental health, recognizing that repair of solastalgia requires simultaneous intervention at the individual, communal, and institutional levels.

**Abstract:**

It is becoming harder to feel at home. As wildfires, floods, drought, and heat become recurrent rather than exceptional, and as everyday life fills with weather alerts, evacuation warnings, and preparedness drills, many people experience a particular kind of distress: the pain of remaining in a place that is still physically present but no longer feels safe, familiar, or trustworthy. This paper examines that experience through the concept of solastalgia—homesickness felt while still at home—and argues that it is not a private mood or a distortion of thinking, but a meaningful disturbance in the very conditions of dwelling. Bringing together phenomenology, emotional geography, and emotion-regulation theory, it shows how a pervasive “culture of emergency” reshapes the way we inhabit space, time, our bodies, and our relationships with others, narrowing the room we feel we have to live and act. Rather than treating climate-related anxiety as something to be corrected, the paper understands it as an honest response to a world whose stability has genuinely been compromised. It then asks how home might become livable again, arguing that repair must happen at several levels at once—personal, communal, and institutional—and that a workable sense of belonging can be rebuilt without denying what has been lost.

## 1. Introduction

It is becoming increasingly difficult to feel at home. Compared to previous generations, many people today must negotiate multiple, often conflicting, conditions just to secure a stable place of living: coordinating employment in the same geographical area, ensuring that income can sustain rising costs, and, where possible, obtaining housing in locations considered safe. Yet even when these conditions are met, the sense of being “at home” remains fragile. Environmental instability and broader conditions of political, social and environmental insecurity increasingly unsettle the experience of place, limiting the possibility of dwelling with ease, continuity, and trust.

Global environmental change increasingly reorganizes human life not only through physical disruption but through a subtler, pervasive transformation of the affective and cognitive conditions by which the world is perceived and inhabited. Extreme weather events, recurrent wildfire seasons, hazardous air, drought, ecosystem degradation, and landscape instability do not simply *happen* to subjects; they recalibrate how threats are detected, how attention is distributed, how risk is evaluated, how uncertainty is interpreted, and how practical decisions are made in the texture of ordinary days. What is striking, however, is that this reorganization now occurs not only through direct exposure to disaster but also through the mediated atmospheres of anticipation and warning that accompany environmental volatility.

Across diverse climate-exposed regions—such as the fire-prone landscapes of the western United States and Australia, flood-affected river basins in South and Southeast Asia, and heat-stressed urban corridors in Southern Europe and the Middle East—everyday life is increasingly organized around systems of environmental alert. Meteorological notifications—heat advisories, red-flag fire warnings, air-quality alerts, flood watches, and updates on large-scale phenomena such as “atmospheric rivers” or monsoon surges—now arrive with a frequency and visual intensity once associated with exceptional crises. The semiotic form of “breaking news,” historically reserved for rare and urgent events, has migrated into routine environmental communication, contributing to a chronic sense of emergency that is no longer episodic but ambient.

This shift generates an affective paradox. While alerts are designed to inform and protect, their continual repetition can foster a state of anxious vigilance, diffuse precarity, and an experience of life as persistently provisional—subject to interruption, cancellation, evacuation, or constraint. Temporal experience is correspondingly reconfigured: rather than unfolding as a continuous horizon for planning and projection, time is apprehended through conditional and contingent intervals (“if the winds shift,” “if the smoke returns,” “if the heat index rises”). The near future thus appears less as an open field of possibility than as a threshold structured by anticipatory risk.

This normalization of the exceptional resonates with Byung-Chul Han’s (2015) critique of contemporary societies that operate through intensifying regimes of urgency, in which emergency becomes a governing modality rather than an exception [1]. In such a view, the constant production of alarm does not merely communicate information; it shapes subjects—cultivating a vigilant, reactive orientation, compressing temporal depth, and weakening the patient forms of attention through which stable meaning and shared worldliness are sustained. In the climatic context, this “society of emergency” is amplified by the fact that the danger is both real and difficult to localize: it arrives as diffuse atmospheric instability, as probabilistic modeling, as alerts that can be simultaneously necessary and exhausting. The atmosphere becomes ethically and existentially charged: not only the weather outside, but the felt “weather” of consciousness—an anticipatory tension, an unsettled readiness, a background fear without a clearly bounded object.

The notion of solastalgia offers a precise name for a specific quality of distress within this affective regime: the pain of remaining “at home” while one’s home becomes unrecognizable, unsafe, or ethically compromised (Albrecht et al., 2007; Galway et al., 2019) [2,3]. Solastalgia is not only an emotion but a disturbance of place—a crisis in the structure that sustains belongingness, meaning, and interpersonal life. Importantly, the contemporary alert ecology intensifies this disturbance: it does not merely report changes in place; it *keeps place in suspense*. One can remain physically situated while being affectively displaced—living in a home that is continuously framed as a potential hazard zone, a site of imminent evacuation, a region under watch. In what follows, I develop this claim in two steps. In the first part, I reconstruct the phenomenon of solastalgia and clarify the central role of place. In the second part, I show how a phenomenological emotional geography can describe the way solastalgia reorganizes the experienced world—how it transforms space, time, bodily attunement, and the sense of possibility.

My aim is to articulate solastalgia as an eco-affective cognitive configuration: a way the world is disclosed when environmental change becomes ambient, recurrent, and only partially controllable. Under such conditions, reality is experienced not simply as altered, but as precarious; attention becomes saturated by threat cues; uncertainty is amplified; and the practical horizon of ordinary action contracts. The “alerted” world is a world in which the ordinary is continuously shadowed by the exceptional, where the future is approached under the mode of contingency, and where the question is no longer merely *what shall we do today* but *will today remain doable*.

The contribution of this paper is therefore conceptual rather than empirical, and its method is interdisciplinary by design. Existing research (Albrecht, 2005; Albrecht et al., 2007; Askland & Bunn, 2018; Galway et al., 2019; Higginbotham et al., 2006) [2,3,4,5,6] has established, through survey and ethnographic work, that solastalgia is real, widespread, and measurable; what remains comparatively underdeveloped is an account of its internal structure—of how, at the level of lived experience, the loss of solace reorganizes a person’s relation to time, space, body, and others. To articulate that structure, the paper reads solastalgia along three converging axes: a phenomenological axis (drawing on Husserl, Merleau-Ponty, and generative phenomenology) that analyzes the constitution of home, lifeworld, and the breakdown of background trust; an emotional-geography axis that treats affect as a publicly and materially conditioned mode of world-disclosure rather than a private interior state; and an emotion-regulation axis that reframes climate-related distress not as faulty cognition to be corrected but as a faithful registration of a genuine structural predicament. The novelty of the approach lies in holding these three registers together and in adding to them the analysis of a contemporary “culture of emergency” that intensifies solastalgia below the threshold of reflection. A philosophical and existential treatment is necessary here precisely because the phenomenon resists the individualizing frame of clinical pathology: it concerns the conditions of dwelling themselves, which are shared, emplaced, and intersubjective. The payoff is both theoretical and practical. Theoretically, it supplies a vocabulary for what empirical instruments measure but do not explain. Practically, it has consequences for how climate-related distress is addressed in clinical, community, and policy settings: if solastalgia is a world-relation problem rather than a private dysfunction, then restoration must operate at several scales at once—individual practices that widen lived leeway, communal rituals and mutual aid that hold grief collectively, and institutional and environmental-justice measures that stabilize the material bases of ordinary life.

### 1.1. Solastalgia at Home

Solastalgia is a neologism coined by the environmental philosopher Glenn Albrecht to name a distinctive form of distress produced by environmental change: an “anxiety of displacement without leaving,” a homesickness for a home that might still exist geographically but no longer exists *experientially*. A variety of factors can trigger solastalgic distress: extreme events (flooding, wildfires, earthquakes) that abruptly remake environments (Warsini, 2014) [7], chronic processes such as drought and sea level rise, resource extraction and industrial degradation (Canu, 2017) [8], climate change as a compounding driver (Tschakert et al., 2013) [9], and political violence that makes homes uninhabitable (Sousa, 2014) [10]. These triggers can be acute (sudden destruction) or chronic (gradual unmaking). The vocabulary often used—“cumulative,” “compounding,” “unwelcome,” “imposed,” “profound”—reveals how environmental change is lived not merely as transformation but as destabilization of the conditions of dwelling (Galway, 2019; Albrecht, 2005, 2010; Pannell, 2018) [3,4,11].

Although the analysis offered here is primarily phenomenological, the phenomenon it describes has been documented empirically across a wide range of settings, in both the Global North and the Global South, and these studies lend concrete texture to the lived experience under discussion. In the open-cut coal-mining districts of the Upper Hunter Valley in New South Wales, Australia, Connor et al. (2004) [12] recorded residents’ sense of desolation as a valued landscape was progressively unmade, the case from which Albrecht first developed the concept. In southeast Ireland, Phillips and Murphy (2021) [13] found that close to half of a coastal community surveyed reported solastalgia following the slow loss of a local beach to coastal erosion, with distress most acute among long-term residents and positively associated with place attachment. In Indonesia, Warsini, Mills, and Usher (2014) [7] traced solastalgic distress among survivors of volcanic disaster, while in the rural and migrant communities of northern Ghana, Tschakert, Tutu, and Alcaro (2013) [9] documented the embodied grief and loss of belonging that accompany incremental climatic and environmental change among populations whose livelihoods depend directly on the land. Scoping and empirical reviews of this literature confirm that solastalgia is now evidenced across diverse geographies and social positions, even as the experience is inflected by local conditions of vulnerability, dispossession, and inequality (Galway et al., 2019; Rafa et al., 2025) [3,14]. These findings are relevant for the present argument in two ways: they confirm that solastalgia is not a marginal or idiosyncratic mood but a widely shared response to environmental destabilization, and they show that its severity tracks not only the magnitude of physical change but the depth of attachment and the degree of social and material precarity through which that change is borne.

In Albrecht’s own formulation, solastalgia is “the pain or sickness caused by the loss or lack of solace and the sense of isolation connected to the present state of one’s home and territory” (Albrecht, 2005, p. 45) [4]. From a phenomenological perspective, this distinction resonates strongly with Steinbock’s (1995) account of the *homeworld* (Heimwelt) and the *alienworld* (Fremdwelt) [15]. For Steinbock, the homeworld is not merely a physical locale but a normative and affective horizon in which meaning is familiar, tacitly shared, and experientially sedimented. It is the dimension in which one’s practices, expectations, and relations unfold with a sense of obviousness and trust. By contrast, the alienworld is not simply “elsewhere” or geographically distant; it designates a mode of appearing in which what was once familiar becomes strange, opaque, or resistant to habitual sense-making. Crucially, the alienworld can emerge *within* the homeworld itself, not only at its borders.

Read in this light, solastalgia can be understood as a disturbance in the very constitution of the homeworld: the place that once functioned as a stable horizon of familiarity begins to take on the characteristics of an alienworld. The transformation is not merely cognitive or representational but affective and embodied. What was previously lived as a background of ease—where one could dwell without vigilance—becomes charged with unease, unpredictability, and anticipatory tension. The erosion of environmental stability thus precipitates a kind of internal estrangement: one remains spatially “at home,” yet experientially displaced.

Albrecht emphasizes that solastalgia differs from nostalgia because it is not oriented toward a distant past but toward an *injured present*: it arises when “the place where one resides and that one loves is under immediate assault,” attacking sense of place and belonging and producing distress about transformation (Albrecht, 2005, p. 45) [4]. Crucially, he also notes that solastalgia can be triggered not only by slow degradation but by acute events—“drought, fire and flood” being among the forces that can provoke it (Albrecht, 2005, p. 46) [4]. Phenomenologically, this means that solastalgia is not only an emotion “about climate change” but a reorganization of the lived structure of home. In Steinbock’s terms (2003) [16], it marks a shift in the homeworld’s internal coherence, as elements of the alienworld intrude and destabilize its taken-for-granted unity. Home becomes less a horizon of shelter and more a site of anticipatory threat—no longer the place where the world holds together, but where it begins to come apart.

### 1.2. State of Emergency

Another issue adds to what presented above. Even when the house is still standing, the mind is increasingly occupied by scenario-building: catastrophes in which everything can disappear “in a moment.” In my own case—living along the San Antonio Fault in California—I fall asleep most nights running through the location of emergency kits and mentally rehearsing the sequence of steps I am supposed to take if the ground moves. That nightly rehearsal is not simply a private habit; it is a socially distributed discipline. California public schools are required to maintain earthquake emergency procedures and training as part of school safety planning (California Education Code §32282, n.p.) [17], and the California Department of Education explicitly notes that schools can meet earthquake drill requirements through participation in the Great California ShakeOut (CDE, 2025 letter, n.p.) [18]. From early childhood through adulthood in workplaces, preparedness is routinized as a pedagogy of catastrophe.

Preparedness can be rational and ethically responsible—and yet its repetition has an affective cost: it can install a background ontology in which the world is experienced as only conditionally stable. Here Byung-Chul Han helps sharpen what is at stake. In his interview “COVID-19 Has Reduced Us to a ‘Society of Survival’” (reprinted in *Capitalism and the Death Drive*), Han argues that fear tends to normalize exceptional measures: crisis “establishes the state of emergency as normal” (Han, 2021, p. 120) [1]. He describes a “survival society” in which survival becomes absolute, “as if we were in a permanent state of war,” and in which the horizon of the good life contracts under the pressure of fear (Han, 2021, p. 121) [1]. Transposed into an ecological register, this is not simply a metaphor: the permanent emergency is lived through fire seasons, heat warnings, flood risk, seismic drills, and the intimate work of nightly rehearsal. The result is a specifically solastalgic impairment of dwelling: the inability to feel safe at home, even when one is still there.

This impairment becomes even more concrete when security is undermined at the institutional level. In California, wildfire risk has been strongly linked to instability in the homeowners insurance market—insurers halting new policies, coverage becoming harder to secure, and growing reliance on the FAIR Plan (the California FAIR Plan, or “Fair Access to Insurance Requirements” Plan, is the state-created not-for-profit insurer of last resort, which provides basic fire coverage to property owners who cannot obtain it on the standard market), alongside regulatory efforts to require insurers to expand coverage in high-risk areas. Whatever one’s policy interpretation, the experiential implication is clear: when coverage becomes uncertain or unattainable, the home loses one of its modern guarantees of recoverability. Home is no longer experienced as a protected continuity but as a precarious asset exposed to abrupt loss.

Taken together, these elements clarify how solastalgia is not merely sadness about environmental change; it is the lived discovery that home no longer functions as home. The ecological (and meteorological) state of emergency does not only threaten the material world; it colonizes the background of consciousness, saturating attention with catastrophic possibility and replacing repose with rehearsal. In this sense, solastalgia names a crisis of inhabitation itself: a form of being “at home” in which one can no longer *arrive* there.

## 2. Solastalgic Anxiety and the Notion of Place

There is a Sicilian saying, “*Cu nesci, arrinesci*”, that loosely translates as “Who leaves succeeds.” Sometimes life, for one reason or another, pushes us away from our place: from what we are accustomed to, from the landscapes and spaces that feel familiar to us. The proverb appears at the beginning of a story about the Florio’s family living in Calabria after the devastating earthquake that forced them to leave their hometown and seek a new life in Sicily where they built the flourishing enterprise that would later make their Marsala wine famous throughout the world.

Finding our place in the world is often tied to a fleeting moment that reshapes our lives, demanding both courage and immense effort. We can never truly feel at home unless we find a place to which we belong, a place where the feeling of home can take root. Yet it is extremely hard to find a place that is entirely free from suffering or uncertainty: our sense of solastalgic anxiety and powerlessness often makes the place we inhabit feel even smaller and more fragile. Place, in the phenomenological sense, is never a neutral backdrop. Jean-Luc Nancy describes it as the space of community or being-with—“an opening of being to itself as common space” (1993, p. 78) [19]. Trujillo (2009) adds to this: place is not an object for detached appreciation but a world-structure that holds together practices, relations, and forms of living [20]. Where outsiders may perceive “space-landscape,” those who dwell within a place experience it as lived continuity (Trujillo, 2009, p. 12) [20]. Environmental degradation, then, is not damage “out there.” It is an assault on the very conditions that make life coherent. The anxiety and the emotional damage we experience shrinks our place and we become a vortex of feelings that often pushes out to venture and try a better life elsewhere.

In solastalgia, this collapse is not only ecological but existential, social, and intersubjective. Place is endangered precisely insofar as it exceeds landscape: it is the structure that binds lives together materially and meaningfully—and this binding is always already a *shared* binding. Husserl understood the lifeworld (*Lebenswelt*) not as a solitary horizon but as a communal one, constituted through what he calls “empathy” (*Einfühlung*) and the ongoing work of intersubjective “harmonization” (*Cartesian Meditations*, §54–55) [21]: we inhabit place *together*, and our sense of home is co-constituted through shared orientations, shared memories, and shared futures held in common.

When environmental anxiety takes hold, it is precisely this intersubjective texture of place that begins to unravel. We no longer drive across town to visit a friend—not because the distance has changed, but because the air quality index has. We no longer send our children to play in the park they have always played in, the one where they know every corner, every other child’s name. We no longer plan the trip we have talked about for years, because we cannot trust that the landscape will still be there, or that it will be safe to cross. These are not merely private losses. They are ruptures in what Husserl calls the *Umwelt*—the surrounding world constituted through shared bodily orientation and habitual co-presence with others (*Ideas II*, §50–51) [22]. Each cancelled visit, each foreclosed plan, each space abandoned to fear is a small amputation of the intersubjective world.

This endangerment operates through three interlocking mechanisms. First, anxiety itself narrows the perceptual field, shrinking one’s capacity to inhabit or appreciate a place—and narrowing, too, one’s felt sense of who else belongs to that place, whose life intersects with one’s own there. Second, what we called above a “culture of emergency”—alerts, preparedness drills, institutionalized crisis protocols—compounds this pressure, further contracting the felt space of dwelling into something managed and provisional rather than open and shared. Third, the concrete triggers of environmental disruption—wildfire, flood, toxic air—can make a place disappear altogether, at least as a source of continuity and shelter. And when place disappears, it takes with it the intersubjective infrastructure that depended on it: the spontaneous encounters, the habitual routes, the common ground—in both the literal and the philosophical sense—that made a community more than a collection of isolated subjects.

What is lost, then, is not only a landscape but a *we*. Husserl insisted that the constitution of the Other is never complete in a single act of empathy but is ongoing, sustained through shared temporal and spatial horizons (*Cartesian Meditations*, §55) [21]. Solastalgia, understood intersubjectively, is the attrition of those horizons—the slow or sudden subtraction of the places where *we* was possible.

For a solastalgic person, emotions attached to place function like what Sartre called a “magic wand,” evoking a world that appears bleak, overwhelming, or unreal—a “world of dreams or madness” (Sartre, 1939/1996) [23]. The distressed person becomes the subject of a passive intentional experience that collapses linear time and compresses space: one is returned to the affective point of disaster not through cognitive recollection alone but through atmospheric cues, smells, seasonal rhythms, and bodily sensations. This passivity is not dysfunction but structure—the structure of affect as a mode of world-disclosure.

Tillich (1952) [24] offers a further conceptual resource: anxiety, he argues, is rooted in finitude and crystallizes around concrete threats to integrity, above all the threats of losing time and losing space. Tillich’s mid-century account can be brought into dialogue with more recent phenomenological work on the temporality of climate-related loss. Hepach and Hartz (2023) [25], drawing on Heidegger, Watsuji, and Waldenfels, argue that climate change has a distinctive temporality in which the loss of a familiar world is registered as a change in the very “referral nexus of significance,” so that place becomes existentially unfamiliar even as it remains physically present—a disruption of lived time and place that exceeds traditional accounts of economic or non-economic loss. Under conditions of environmental destabilization, both dimensions are directly imperiled. Spaces become unsafe, inaccessible, or ethically compromised; time becomes erratic as seasons destabilize and the future is experienced less as a horizon of open projects than as a sequence of risk-scenarios and possible disruptions. The culture of emergency intensifies this dynamic. Environmental instability becomes not only an objective condition but a mediated and institutionalized atmosphere: the normalization of “emergency” recalibrates the background of consciousness so that catastrophe becomes continuously thinkable, continuously near, and therefore continuously present as a shadow. Solastalgic anxiety is thus not best understood as an internal malfunction. It is an intelligible response to a world whose stability has been compromised and whose future has become uncertain in an ongoing, ambient way—what we might call a structurally induced vigilance rather than a disproportionate reaction.

## 3. Emotions and Reality

Emotions often have the power to *make* reality salient—not by fabricating facts, but by reorganizing the very field in which facts can matter. Phenomenologically, “reality” is never merely a collection of objects existing “out there”; rather, it is the world as it appears, together with the affective-intentional structures through which it is experienced as threatening, safe, urgent, familiar, or provisional. Emotions are therefore not merely inner events hovering above the world. They are concrete orientations that redistribute attention, weight meanings, and open or close horizons of possibility. In this sense, it can indeed be harder to “walk away” from an emotion than from a room—because the room can be left, while the affective attunement continues to *hold* the world in a particular profile of significance.

This becomes especially legible under conditions of environmental instability and the contemporary culture of emergency. When meteorological volatility, wildfire seasons, hazardous air, drought, and seismic risk are no longer exceptional but recurrent, the background trust that ordinarily supports dwelling is weakened. The constant circulation of alerts and preparedness scripts amplifies this weakening by producing a permanent anticipatory stance: attention is repeatedly pulled toward worst-case scenarios, and the near future is lived as a sequence of conditional threats. Crucially, many of these threats are not hallucinated: they are plausible, modeled, warned, rehearsed. Yet their omnipresence in experience can install a second layer of “world” inside the first: a catastrophic world that continuously *prefigures* the everyday one, so that home is inhabited under the tacit condition that it might cease to exist “in a moment.”

Husserl’s distinction between the *real* and the *reell* helps clarify what is happening here. In his critique of Brentano’s “immanent object,” Husserl insists that the intended object (Jupiter, Bismarck, a cathedral, a polygon) is not a piece inside consciousness; it is not part of the experience’s “descriptive or real make-up (deskriptiven reellen Bestand).” The experience is real (reell) as a lived act, but the object “naturally will not be found in it” (Husserl, *Logical Investigations*, 2001, pp. 558–559) [26]. Put differently, the *reell* names the immanent constituents of experience—sensations, affective tones, noetic enactments—whereas the *real* in the ordinary sense concerns what is transcendent to the act (the thing, event, or state of affairs intended). Husserl’s point is not that the world is unreal, but that the world’s appearing is structured by components that are not themselves “objects out there,” even though they decisively shape what counts as urgent, dangerous, or livable.

This is precisely why emergency culture has such phenomenological force. Alerts, drills, and rehearsals do not merely convey information about the *real*; they sediment into the *reell* texture of consciousness—into background vigilance, anticipatory fear, and scenario-thinking—thereby reshaping world-disclosure. Environmental instability supplies the plausibility conditions; emergency media and institutional preparedness supply the cadence; together they can produce an affective regime in which catastrophe is continuously “co-present” with the everyday. The home remains *real* and geographically present, but it is lived through *reell* structures that withhold full security and belonging. The result is not a private mood added onto an unchanged world; it is a transformed lifeworld in which the familiar is continuously shadowed by the possibility of sudden loss.

If affects are understood as “a transpersonal capacity which a body has to be affected (through an affection) and to affect (as the result of modifications)” (Anderson, 2006, p. 735; see also McCormack, 2003; Thrift, 2004a) [27,28,29], then the analytic question becomes more precise: what does our place look like, feel like, and allow, when environmental instability and emergency culture sediment into the *reell* life of consciousness? And the practical question follows immediately: how might this affective place be made livable again—how might the home-world recover enough trust, temporal depth, and shelter to be inhabited as home?

### 3.1. Emotional Geography of Anxiety

To address these questions we can look at how emotional geography handles the problem of anxiety. Emotional geography often describes anxiety in the shape of a black hole. Anxiety draws individuals into an atemporal affective space saturated by sensations and meanings not yet metabolized. The triggering object functions like a temporal wand that reopens the spatiality of trauma. The anxious world is not merely “the same world plus fear.” It is a world whose distances, thresholds, and significances have been transformed, to use Husserl’s vocabulary, at the level of the life-world (*Lebenswelt*). What was once a stable horizon of implicit trust becomes fractured and volatile. Objects no longer recede into a reliable background but press forward with excessive salience; space is reorganized through lines of attraction and repulsion; temporality loses its flowing continuity and is reconfigured into a thickened present dominated by protentional anticipation of threat. The world feels unstable, intrusive, and conditionally available. It affords not easeful movement but vigilance, monitoring, and withdrawal. Action is no longer guided by sedimented familiarity but by the effort to manage exposure within a field of latent danger.

In *A Sketch for a Theory of the Emotions* (1939/1996) [23], Sartre shows how objects can become triggers for intense fear; anxiety attached to an intentional object transforms space into danger. A place once comforting can become unbearable given its present disruption. People with phobic anxiety describe certain objects as “emotional black holes” stretching and compressing their world. Sartre reports the case of a young woman who fainted at the sight of laurel, later connected through analysis to childhood abuse (Sartre, 1939/1996, p. 50) [23]. Fell remarks that for an anxious person, the environment is perceived as a set of attracting or repelling vectors directed toward or away from objects (Fell, 1966, p. 15) [30].

Geographers in the phenomenological tradition have argued that any existential presentation of the geographical lifeworld must recognize the personal and cultural dimensions of placed experience (Seamon & Sowers, 2009) [31]. The trajectory from affects to sensations to emotions—passive intentionality—gives meaning to the world such that “the emotional subject and the object of the emotions are unified in an indissoluble synthesis” (Sartre, 1996/1933) [23]. As long as this synthesis remains passive, the subject has little distance from the affective world that overwhelms them. For this reason, geographers in human geographical research have long treated emotion not as a subjective residue but as a mode of situated knowing and doing. A major shift occurred when Anderson and Smith argued for recognizing emotions as ways of knowing, being, and acting that extend geographical understanding beyond predominantly visual or textual registers (Anderson & Smith, 2001) [32]. Subsequent work in emotional and affectual geography developed along multiple lines, including technologically mediated affect worlds (Shaw & Ward, 2009; Budd & Adey, 2009) [33,34], the naming and meaning of places (Kearney & Bradley, 2009) [35], and lived experiences of pain (Bissell, 2009) [36]. Within this framework, solastalgia can be illuminated not as a “private feeling” but as a *publicly and materially conditioned* mode of world-disclosure: a way the world appears when the stability of place is compromised and the affective background of dwelling is reorganized.

In this context, interpersonal support can make a difference because it offers an intersubjective alternative reality that can hold the person while meanings are reorganized. Technology-mediated connection can help, but what is crucial is not the medium—it is the restoration of shared space as a horizon of meaning. The absence of social support to address the practical and emotional consequences of displacement and degradation severely impacts well-being. As one testimony in Albrecht’s mining research expresses: “There’s no social support for displacement, none whatsoever” (Lea, cited in Albrecht, 2005, p. 52) [4].

Emotions themselves are environmental. We cannot choose when an emotion will arise or how long it will last; it “fades away of itself,” but cannot be stopped by will alone (Sartre, 1996/1939, pp. 75–76) [23]. Emotion is concrete like brick walls: it is a lived structure one must enter in order to find the door. When affects elicit anxious reaction, geographical distance disappears because the person’s world collapses into the trigger. In solastalgia, the trigger is the space with which the person has identified themselves. Time becomes non-linear; the ordinary categories of the world lose their stability (Sartre, 1996/1939, p. 90) [23].

This structure becomes crucial for understanding solastalgia. When the “trigger” is not a discrete object but the very fabric of place—air, landscape, seasonality, water—the disturbance penetrates the horizonal structure of experience itself. Environmental instability and emergency culture sediment into passive syntheses, reshaping how the world is pre-reflectively constituted. The horizon no longer functions as an open field of indeterminate possibility but as a saturated field of anticipated disruption. Protention is colonized by scenarios of loss, while retention loses its stabilizing force as past regularities no longer guarantee future continuity. The home-world, in this condition, is no longer given as a sheltering ground but as an unstable and demand-laden field that requires continuous affective regulation. One does not simply dwell; one manages one’s dwelling.

Merleau-Ponty uses the notion of *Spielraum* (leeway) to describe the lived space of maneuver that makes action and meaning possible (Merleau-Ponty, 2012) [37]. Spielraum describes a range of possibilities that move outward from an enticement and guide individuals to fulfill anticipations with new perceptual contents (Husserl, 2001, 87; Moran, 2005, 162–3) [26,38]. In solastalgic anxiety, Spielraum contracts. The field of possible action narrows and becomes heavy with consequence. The world ceases to offer a generous horizon of ordinary possibilities and becomes a field of constrained choices.

### 3.2. Regaining Our Home in Our Place

The preceding analysis has traced a descent: from the general conditions of environmental instability and emergency culture, through the phenomenological structure of solastalgia as a disturbance of place, to the emotional geography of anxiety as a contraction of the affective world. What remains is the question the paper has been moving toward all along—not diagnostic but restorative. If solastalgia reorganizes the lived field in which subject and world co-emerge, the task is not merely to name that reorganization but to ask how it can be undone, or at least moved through: how the place we inhabit in the world, often marked by instability, can become livable enough again for a house to be felt as our home, and for a place to be inhabited rather than merely endured.

The first condition of any answer is that the distress be taken seriously on its own terms. A more adequate model of emotional regulation—developed in the work of Mennin and colleagues [39,40]—interprets anxiety not as irrational content to be corrected but as involving heightened emotional intensity, poor understanding of emotions, and maladaptive relationship to one’s own affective states. Friman, Hayes, and Wilson (1998) [41] press this further: painful emotions should not be avoided as a general category, because they are cues for understanding reality. For solastalgic subjects, this is not a minor theoretical point. The distress is not a misreading. It is a faithful, if anguished, registration of a genuine structural predicament—one in which the background trust that sustains dwelling has been genuinely eroded. To treat it primarily as cognitive distortion is not only phenomenologically inaccurate; it risks redirecting attention from shared structural conditions to individual pathologies, compounding the very isolation that solastalgia already installs.

What is needed instead is what might be called compassionate acceptance of the altered world—not resignation, and not the passive endurance of what cannot be changed, but a willingness to encounter painful affect fully enough that it does not become a frozen, recurrent intrusion. Sartre’s account of passive intentionality showed how the solastalgic person is drawn back, again and again, to the affective point of loss through atmospheric cues, seasonal rhythms, and bodily sensations that carry the imprint of what has changed. This passivity is the structure of affect such as world-disclosure; it cannot be bypassed by an act of will. But it can be moved through; when affect is metabolized into meaning rather than merely managed, and when the grief of environmental loss is felt in its depth rather than suppressed, the alterity of the world can begin to reopen: its capacity to surprise and shelter as well as threaten, and to offer more than the saturated profile of danger through which anxiety apprehends it.

This is where Merleau-Ponty’s notion of Spielraum becomes not only diagnostic but generative. The contraction of lived leeway that characterizes solastalgic anxiety is not irreversible. Spielraum can be gradually reconstituted—not by eliminating uncertainty, which the ecological conditions of the present do not permit, but by re-establishing a differentiated relationship to it. Not everything needs to appear equally charged; zones of relative familiarity and safety can re-emerge within a world that remains, at its edges, genuinely precarious. The future can regain something of its character as open—not free of threat, but not reducible to threat either. Trust is not restored as a belief about what will happen. It is restored, as Husserl understood, as a pre-reflective certainty grown from repeated, embodied encounters with a world that, despite its instability, continues to hold in some respects—through rhythms, textures, presences, and small confirmations that accumulate below the level of reflection.

At the individual level, this involves practices that widen rather than further contract Spielraum: naming emotions rather than suppressing them, recognizing triggers without remaining entirely captive to them, reducing avoidance where it is safe to do so, and building tolerance for uncertainty while sustaining a sense of agency. These are not merely coping techniques. They are ways of re-inhabiting the world—of allowing the lifeworld to re-sediment, gradually, into something that can again function as background rather than foreground, as support rather than threat. The restoration of livability therefore requires a transformation at the level of this affective-horizonal constitution. The home-world becomes inhabitable again when passive syntheses begin to sediment differently—when regularities, rhythms, and patterns of reliability are re-established in lived experience. This involves the gradual reconstitution of temporal thickness, in which retention can once again support protention, allowing the future to appear not only as threat but as open possibility. It also requires the re-stabilization of spatial horizons, such that not everything appears equally charged or urgent, and zones of relative safety and familiarity can re-emerge. Trust is not restored as a belief but as a pre-reflective certainty grounded in repeated, embodied encounters with a world that holds. Through this re-sedimentation, the Lebenswelt regains its function as a background of support rather than a field of constant disruption. The world becomes livable again not because uncertainty disappears, but because it is re-integrated into a differentiated horizon in which dwelling, attachment, and meaningful action are once more possible.

But the reconstitution of dwelling cannot remain at the individual level—and this is perhaps the most important claim the paper’s analysis has prepared. Place, as the preceding sections argued, is never constituted in solitude. It is always already a shared structure, co-constituted through intersubjective harmonization, shared orientations, shared memories, and futures held in common. What solastalgia puts at risk is not only one person’s sense of belonging but also the intersubjective fabric through which belonging becomes possible at all. What is lost, as we saw, is not only a landscape but a *we*. Repair must therefore also be communal.

At the collective level, regaining our home in our place involves rebuilding place as shared: through mutual aid and community resources that restore practical trust, through culturally meaningful rituals of loss and restoration that allow grief to be publicly held rather than privately borne, and through forms of collective efficacy that counter the helplessness that ambient emergency installs. Where the environment threatens to become what Sartre might call an affective black hole—collapsing temporal depth, compressing space, evacuating the future of its openness—intersubjective life must become the counter-horizon: not by reversing what has been ecologically altered, but by reconstituting, at the level of shared practice and communal form, the conditions under which changed places can once again be inhabited as home.

Hope, understood in this way, is not optimism without evidence. It is a practical possibility sustained by shared structure—the kind of hope that does not deny what has been lost but discovers, within loss, the conditions of renewed beginning. The Sicilian proverb with which this paper’s analysis of place began—*cu nesci, arrinesci*, who leaves succeeds—speaks to the courage demanded by displacement. But the paper’s argument points in a complementary direction: sometimes the more demanding act is to re-inhabit what anxiety has made strange. The home-world becomes inhabitable again not when uncertainty disappears but when it is re-integrated into a differentiated horizon in which dwelling, attachment, and meaningful action are once more possible. The world becomes livable not by reverting to what it was, but by becoming—through effort, practice, and shared presence—somewhere one can, once again, genuinely arrive.

## 4. Conclusions

This paper has argued that solastalgia is not simply an individual reaction to climate change but a disturbance in the very conditions of dwelling—a loss of solace within ongoing habitation that erodes the background trust, temporal depth, and intersubjective fabric through which a place becomes home. The culture of emergency intensifies this erosion below the threshold of reflection: alerts, preparedness drills, and crisis scripts sediment into vigilance and anticipatory tension so that catastrophe becomes continuously co-present with ordinary life. Solastalgic anxiety is therefore not a cognitive distortion or maladaptive appraisal. It is a structurally induced response to a world whose stability has been genuinely compromised.

This has significant implications for how climate-related distress is understood clinically and politically. When solastalgia is framed primarily as dysfunctional information processing, its ethical and epistemic core is missed. What is needed instead is an integrated framework that holds together affective life, environmental conditions, and social infrastructures of support—one that takes painful emotions seriously as knowledge about reality rather than as symptoms of dysregulation, and that recognizes the political consequences of privatizing what is structurally produced. Restoration is possible, but only if understood at the right level: individually, through practices that widen Spielraum and re-sediment the lifeworld as support rather than threat; collectively, through mutual aid, shared rituals of grief, and forms of efficacy that counter ambient helplessness; and politically, through environmental justice and the institutional will to stabilize the material bases of ordinary life.

The conclusion is both modest and urgent. Coping with solastalgia in ways that protect human and ecosystem health requires treating it as a world-relation problem rather than a private pathology. To make dwelling possible again is not to return to an untouched past—it is to rebuild, under altered conditions, forms of safety, trust, and belonging that can hold. In that rebuilding, the pain named by solastalgia need not remain only a symptom to be managed. It can become what it has always also been: a lucid affective knowledge of what matters, what has been lost, and what must be repaired.

## Data Availability

No new data were created or analyzed in this study. Data sharing is not applicable to this article.
